# Dynamics of Skeletal Status under Optimized Management during Subsequent Pregnancy in Three Women with a History of Pregnancy‐ and Lactation‐Associated Osteoporosis Carrying pathogenic Variants in *WNT1* and *LRP5*

**DOI:** 10.1002/jbm4.10779

**Published:** 2023-06-21

**Authors:** Julian Stürznickel, Sebastian Butscheidt, Michael Amling, Ralf Oheim

**Affiliations:** ^1^ Department of Osteology and Biomechanics University Medical Center Hamburg‐Eppendorf Hamburg Germany; ^2^ Department of Trauma and Orthopaedic Surgery University Medical Center Hamburg‐Eppendorf Hamburg Germany; ^3^ Martin Zeitz Center for Rare Diseases University Medical Center Hamburg‐Eppendorf Hamburg Germany

**Keywords:** EARLY‐ONSET OSTEOPOROSIS, FRACTURE, HEREDITARY BONE DISORDER, PREGNANCY‐ AND LACTATION‐ASSOCIATED OSTEOPOROSIS, TREATMENT

## Abstract

Pregnancy‐ and lactation‐associated osteoporosis (PLO) is a rare but clinically highly relevant condition, characterized by reduced bone mineral density (BMD) and acute onset of severe pain due to symptomatic bone marrow edema of the hip or vertebral and/or insufficiency fractures, among others. Previous reports showed a high frequency of hereditary bone disorders unmasked by PLO, predisposing for more severe forms. To date, no data on the risk for additional fractures during subsequent pregnancy in women with PLO and genetic bone disorder have been available. To address this question, we retrospectively analyzed the clinical, biochemical, and densitometric course of three women with a history of PLO and detected variants in *WNT1* or *LRP5* and subsequent pregnancies. Calcium homeostasis and bone turnover were optimized by basic treatment, and timely initiation of weaning was recommended. Teriparatide treatment for 12 months under strict contraception was initiated in one woman after the diagnosis of PLO. In none of the women did additional fractures or symptomatic bone marrow edemas occur, and BMD by dual‐energy X‐ray absorptiometry as bone microarchitecture by high‐resolution peripheral quantitative computed tomography remained stable. In conclusion, this report expands the understanding of this rare but severe condition and helps to improve clinical counseling and management. © 2023 The Authors. *JBMR Plus* published by Wiley Periodicals LLC on behalf of American Society for Bone and Mineral Research.

## Introduction

Pregnancy‐ and lactation‐associated osteoporosis (PLO) is a rare but clinically highly relevant condition. Normal pregnancy and subsequent lactation are known to rapidly reduce areal bone mineral density (aBMD), which is usually restored after completion compared to preconception.^[^ ^1^ ^]^ However, in PLO, physiological changes, including bone loss, are accompanied by pathological findings, such as an acute onset of severe pain ante‐ or postpartum due to excessive bone marrow edemas (BMEs) or vertebral and insufficiency fractures, respectively.^[^ ^2^ ^]^ Potential clinical manifestations include, for example, single or multiple vertebral fractures and BMEs of the sacrum, femur, or tibia that might develop into insufficiency fractures, substantially limiting the quality of life.^[^ ^3^ ^]^ Though considered rare, PLO may often remain undiagnosed as indications for diagnostics are limited, although musculoskeletal pain is commonly found during pregnancy.

The available knowledge of the condition, especially concerning etiology, is relatively scarce.^[^ ^4^ ^]^ During pregnancy, remarkable processes occur that affect maternal calcium metabolism and, consequently, the skeleton, with an increased transfer of calcium to the fetus that reaches an amount of approximately 30 g by term.^[^ ^2^, ^5^ ^]^ In PLO, calcium transfer is assumed to exceed the individual maternal uptake capability. Depending on the duration of lactation, additional loss of bone mass results in decreasing aBMD^[^ ^6^ ^]^ and bone microarchitecture.^[^ ^7^ ^]^ Recently, we were able to highlight the relevant contribution of hereditary factors among others that might lead to the manifestation of PLO.^[^ ^8^, ^9^ ^]^

The onset of PLO most commonly occurs during the first pregnancy and between the third trimester and 6 months after birth, respectively, while symptoms may appear as early as the second trimester; in addition, there are also reports of a first onset in the fifth pregnancy.^[^ ^10^ ^]^ In premenopausal women, the use of antiresorptive agents is strictly limited and requires adequate contraception. In general, agents that do not bind to bone matrix are preferred, such as teriparatide^[^ ^2^, ^10^, ^11^ ^]^ or denosumab,^[^ ^12^ ^]^ which lead to clinical and densitometric improvements. However, denosumab should only be used in the short term (i.e., once); otherwise, the rebound phenomenon should be taken into account and intravenous bisphosphonates may be indicated, as drug vacations may be critical in these young patients.^[^ ^13^ ^]^

Reports of women with previously diagnosed PLO have shown a relevant risk for additional fractures in general and during a subsequent pregnancy in particular.^[^ ^14^ ^]^ In a report on 30 women with a history of PLO and a subsequent pregnancy, 20% had a recurrence of the disease, that is, fractures. However, no studies in women with PLO and a hereditary bone disorder regarding the risk for recurrence or further fractures during subsequent pregnancies are available. Therefore, we analyzed the clinical, biochemical, and densitometric changes in women with detected pathogenic variants in *LRP5* or *WNT1* during consecutive pregnancies to improve the understanding and clinical management of PLO.

## Materials and Methods

### Study design

**Fig. 1:**
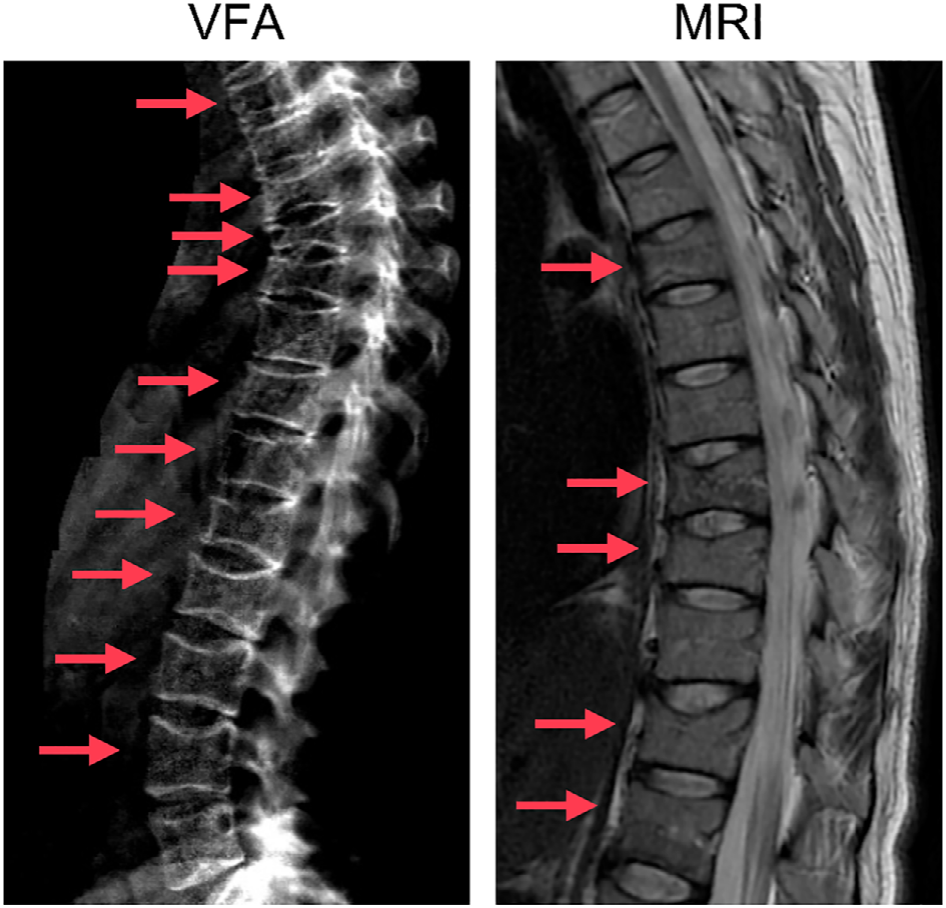
PLO manifestation. Representative vertebral fracture assessment (VFA, left panel) and MRI image of multiple vertebral fractures of two women (left: Individual 3, right: Individual 1) with a history of PLO.

We retrospectively analyzed data from subsequent pregnancies of women who presented to our specialized outpatient clinic due to PLO and were found to have a monogenic bone disorder, as previously reported.^[^ ^8^ ^]^ In short, PLO was defined as an aBMD reduction (Z‐score ≤ −2.0) with occurrence of vertebral fractures, excessive BMEs, or insufficiency fractures (Fig. 1) during pregnancy and up to 6 months postpartum. Patients with a follow‐up of at least 6 months who presented before and after delivery were included. Besides routine examinations, patients had been instructed to contact the department in case of any skeletal pain to potentially initialize diagnostics and treatment, if required. All individuals gave written informed consent, and the local ethics committee approved this study (PV5364).

### Clinical and skeletal examination

Biochemical analysis of calcium and bone metabolism was routinely performed at baseline (BL) and before pregnancy (BP), during pregnancy (DP), and after pregnancy (AP). In that sense, serum levels of calcium, phosphate, vitamin D, parathyroid hormone (PTH), alkaline phosphatase (ALP), markers of bone formation bone‐specific alkaline phosphatase (BALP), and osteocalcin, as well as urinary bone resorption marker deoxypyrodinoline/creatinine (DPD), were measured.

To evaluate a loss of bone mass, aBMD was measured at the onset of PLO (defined as BL) and during follow‐up, defined as either BP or AP, respectively, by dual‐energy X‐ray absorptiometry (DXA, Lunar iDXA®, GE Healthcare, Madison, WI, USA) at the proximal femora and lumbar spine (L1–4), shown as sex‐ and age‐adjusted standard deviations (Z‐score). To further evaluate three‐dimensional bone microarchitecture, high‐resolution peripheral quantitative computed tomography (HR‐pQCT, XtremeCT®, Scanco Medical AG, Brütisellen, Switzerland) was performed according to guidelines^[^ ^15^ ^]^ at the distal tibia and radius following the in vivo settings provided by the manufacturer. The nomenclature was in accordance with the *IOF‐ASBMR‐ECTS* working group.^[^ ^15^ ^]^ For data normalization, parameters were compared to a reference cohort, presented as percentage of the median.^[^ ^16^ ^]^

### Statistical analysis

Statistical analysis was performed by GraphPad Prism (version 8.4.0, GraphPad Software, Inc., La Jolla, CA, USA). Normality distribution of the data was assured by Shapiro–Wilk test. Student's *t*‐test or one‐way ANOVA, and repeated measures with Tukey correction were performed. The level of significance was defined as *p* < 0.05.

## Results

### Case history

Three women were included in the analysis due to subsequent pregnancy, a history of PLO, and pathogenic variants in *WNT1* and *LRP5* (Table 1). Individual 1 was a 31‐year‐old woman in whom PLO manifested with five vertebral and one peripheral fracture during the third pregnancy. Genetic analysis had revealed a *WNT1* c.943 T > G variant, and the subsequent pregnancy was 3 years after onset of PLO. The second woman was 37 years old, and PLO was diagnosed in the first pregnancy due to five vertebral fractures. Genetic analysis showed an *LRP5* c.1265C > T variant, and the subsequent pregnancy was 4 years later. She received teriparatide for 1 year without consecutive antiresorptive treatment after the PLO diagnosis was established. Individual 3 was a 34‐year‐old woman diagnosed with PLO in the context of her first pregnancy due to 10 vertebral fractures. A *WNT1* c.703C > T mutation was detected, and the subsequent pregnancy was 3 years after onset.

**Table 1:** Characterization of study cohort (showing demographics, details about pregnancy history, and fracture information)

Parameter	Total	Individual 1	Individual 2	Individual 3
Demographics				
Age (years)	34.3 ± 3.1	31	37	35
Weight (kg)	62.1 ± 8.9	72.4	57	57
Height (m)	157.8 ± 10.7	169.5	155.5	148.5
BMI (kg/m^2^)	24.9 ± 1.2	25.20	23.57	25.85
Follow‐up (months)	23.5 ± 8.7	16.3	33.2	20.9
Pregnancies				
*n* of total pregnancies	2.7 ± 1.2	4	2	2
Pregnancy with PLO manifestation	1.7 ± 1.2	3	1	1
Period of onset	Postpartum	Postpartum	Postpartum	Postpartum
Time between pregnancies (years)	3.3 ± 0.6	3	4	3
Time until weaning (weeks)	4.0 ± 2.0	4	2	6
Genetics				
Gene		*WNT1*	*LRP5*	*WNT1*
DNA level		c.943 T > G	c.1265C > T	c.703C > T
Protein level		p.(Cys315Gly)	p.(Ala422Val)	p.(Arg235Trp)
Allele frequency		<0.01%	<0.01%	0%
ACMG class		Likely pathogenic (IV)	Likely pathogenic (IV)	Pathogenic (V)
MutationTaster		DC	DC	DC
PolyPhen score		Damaging	Damaging	Damaging
Fractures at onset				
Vertebral	6.7 ± 2.9	5	5	10
Peripheral	0.3 ± 0.6	1	0	0
Additional fractures				
Vertebral	0	0	0	0
Peripheral	0	0	0	0

Abbreviations: ACMG, American College of Medical Genetics; BMI, body mass index; PLO, pregnancy and lactation associated osteoporosis.

In all subjects, PLO had manifested postpartum, and after the diagnosis was made, breastfeeding was stopped, and vitamin D and calcium supplementation were optimized (Table 2). Moreover, teriparatide was given for 12 months under strict contraception in Individual 2. In Individual 1, specific treatment could not be initiated at first because calcium homeostasis was severely disturbed and took months to correct. Thereafter, early DXA monitoring showed markedly improved BMD levels, and therefore specific treatment was not initiated. In addition, the patient refused treatment with bone‐active drugs. At the initial presentation of Individual 3 to our institution, early DXA follow‐up already showed significant improvement, and bone turnover was balanced, so no specific treatment was initiated.

**Table 2:** Individual supplementation of vitamin D and calcium gluconate

Parameter	Total	Individual 1	Individual 2	Individual 3
Vitamin D (IU/week)				
Before pregnancy	25,000 ± 8,660	20,000	35,000	20,000
During pregnancy^a^	21,000 ± 14,000	21,000	35,000	7000
After delivery	24,500 ± 12,619	21,000	38,500	14,000
Calcium gluconate (mg/day)				
Before pregnancy	166.7 ± 288.7	500	0	0
During pregnancy	0	0	0	0
After delivery	0	300	0	0

^a^ Vitamin D was given daily during pregnancy and breastfeeding.

### Biochemical analysis

**Fig. 2:**
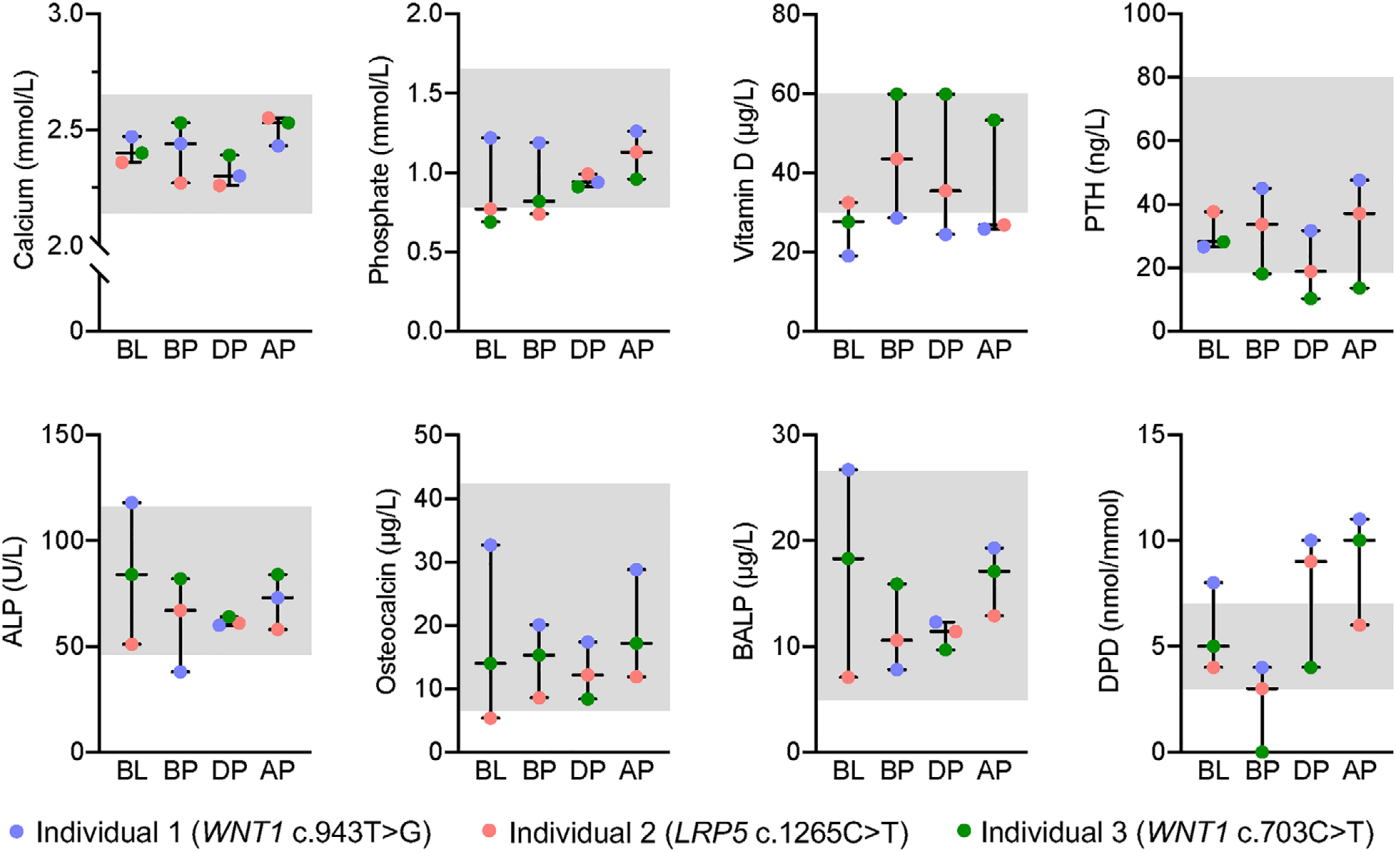
Biochemical evaluation. Biochemical parameters were evaluated after the onset of PLO, BL, BP, DP, and AP. The time interval between the measurements was 12.8 ± 10.0 months (BL to BP), 11.1 ± 4.9 months (BP to DP), and 6.7 ± 2.8 months (DP to AP). The shaded area represents the reference range. ALP, alkaline phosphatase; AP, after pregnancy; BL, baseline; BP, before pregnancy; BALP, bone‐specific alkaline phosphatase; DPD, deoxypyrodinoline/creatinine; DP, during pregnancy; PTH, parathyroid hormone.

After the onset of PLO, serum levels of calcium and phosphate were within or slightly below the reference range (Fig. 2), accompanied by vitamin D deficiency in two out of individuals and PTH levels within the normal reference range. Normal to high bone turnover correlated inversely with vitamin D levels. Before the subsequent pregnancy, vitamin D levels improved in all by supplementation, and bone turnover normalized. During pregnancy, calcium and PTH levels dropped, whereas the bone resorption marker DPD increased above the reference range in two out of the three women within the third trimester (all *p* > 0.05). After pregnancy, the two women with elevated DPD levels were still breastfeeding at the time of biochemical analysis, while the woman with normal DPD levels had already weaned 2 weeks after delivery.

### Bone mineral density, bone microarchitecture, and clinical course after delivery

**Fig. 3:**
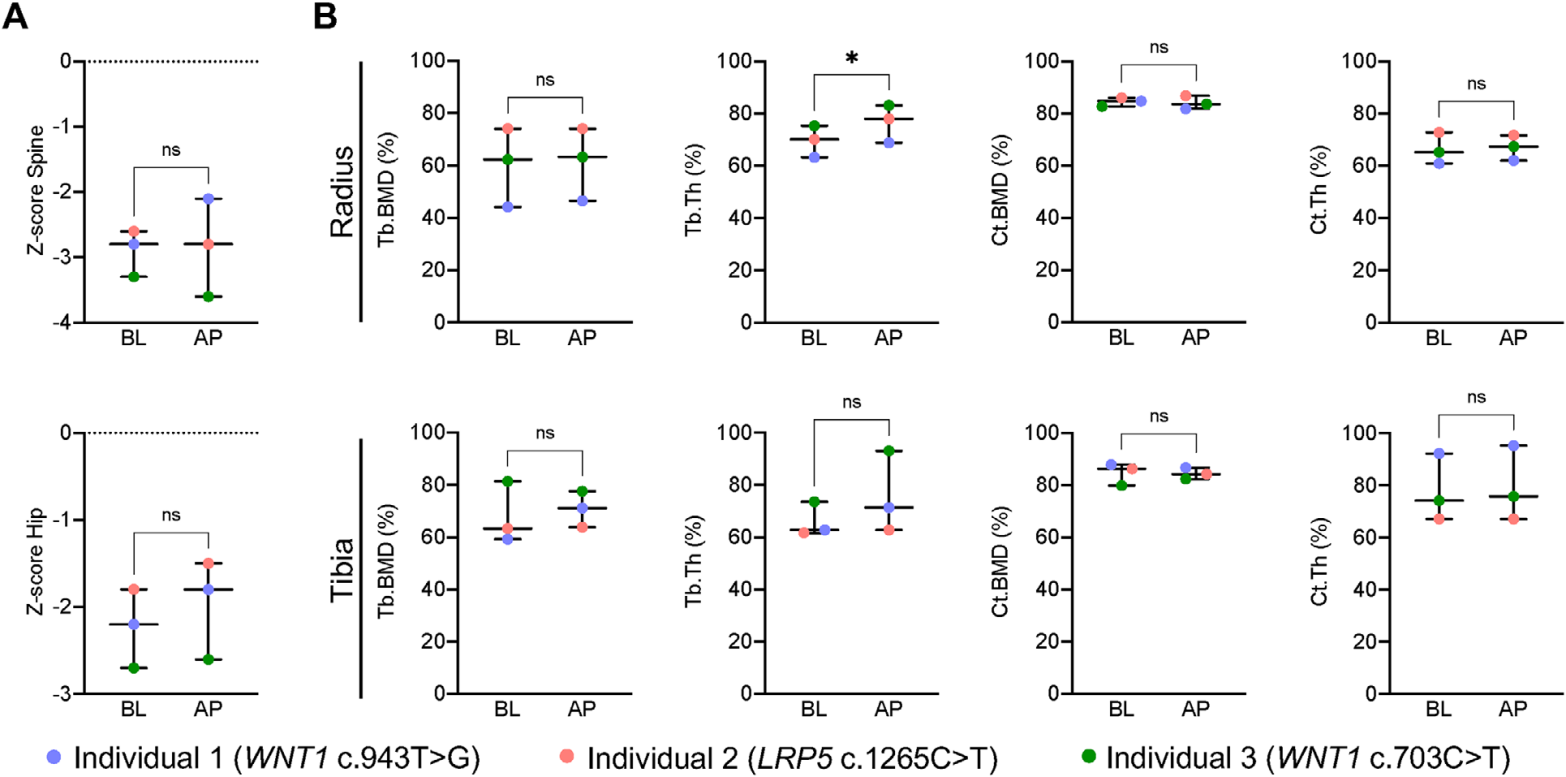
Densitometric evaluation. (A) Areal bone mineral density (aBMD) evaluation by dual‐energy x‐ray absorptiometry (DXA) at lumbar spine and hip. (B) Three‐dimensional evaluation of bone microarchitecture by high‐resolution peripheral quantitative computed tomography (HR‐pQCT) at distal radius and tibia. The time interval between BL and AP was 44, 35, and 17 months for Individuals 1, 2, and 3. *p < 0.05. AP, after pregnancy; BL, baseline; Ct.BMD, cortical bone mineral density; Ct.Th, cortical thickness; Tb.BMD, trabecular bone mineral density; Tb.Th, trabecular thickness; ns, not significant.

Importantly, no aBMD loss was observed after the subsequent pregnancy compared to PLO onset (Fig. 3A). Specifically, one woman showed an increased aBMD Z‐score at the spine (+0.7), and two slightly decreased (−0.2 and −0.3). At the hip, BMD increases were observed in all three women (+0.4, +0.3, and +0.1, all *p* > 0.05). aBMD measurements were obtained 3, 6, and 6 weeks after delivery.

Likewise, the evaluation of bone microarchitecture by HR‐pQCT showed no further deterioration after the subsequent pregnancy (Figs. 3B and S1). Specifically, Tb.BMD, Ct.BMD, and Ct.Th remained unchanged, whereas Tb.Th improved significantly (*p* = 0.011) at the distal radius. At the distal tibia, similar values were observed during the follow‐up examination (*p* > 0.05).

**Fig. 4:**
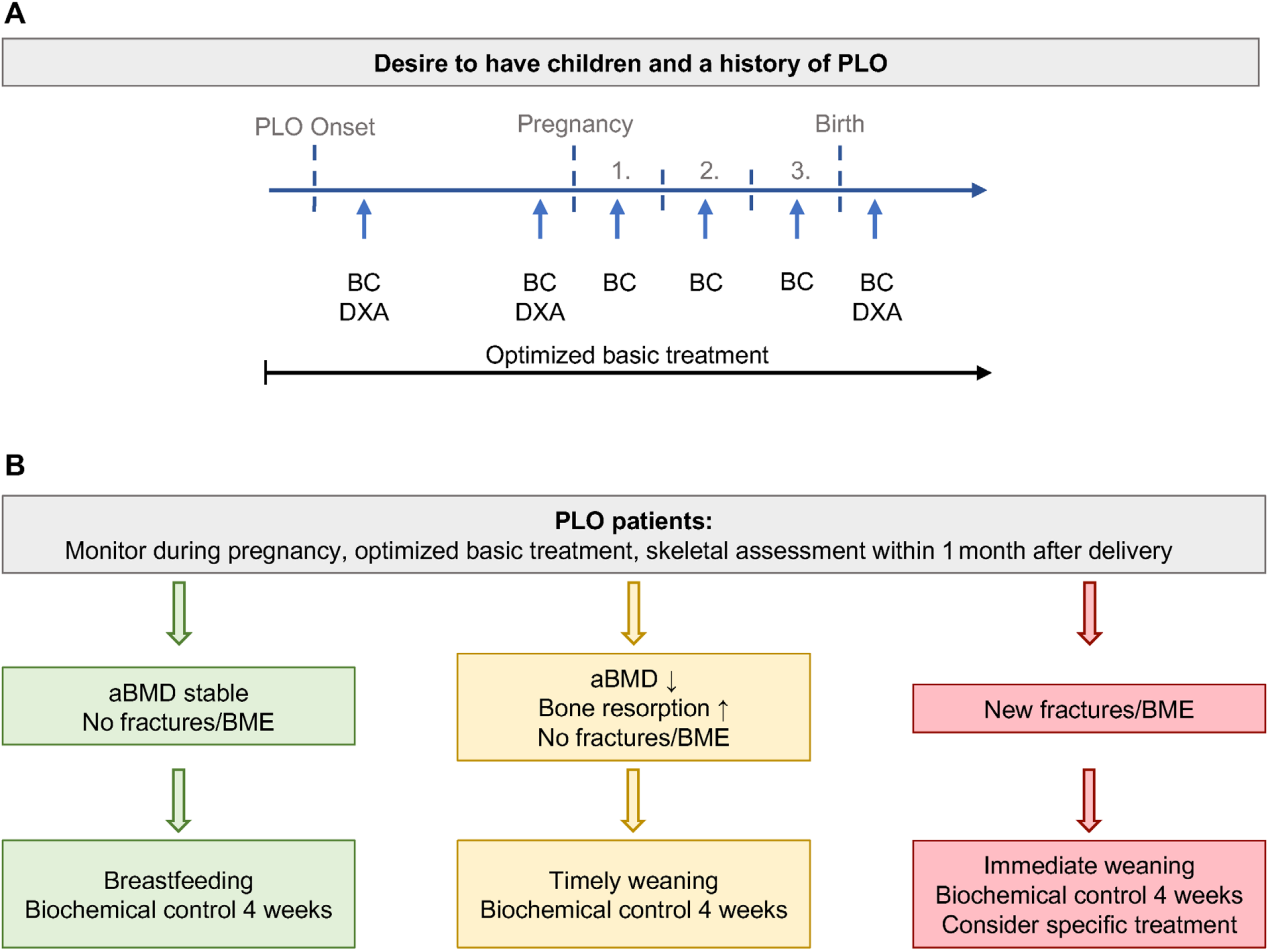
Clinical management algorithm. (A) Proposed monitoring algorithm after delivery in women with a history of PLO. The numbers refer to the respective trimesters of pregnancy in which the check‐ups should be performed. (B) Proposed decision‐making algorithm after delivery. aBMD, areal bone mineral density; BC, biochemical control; BME, bone marrow edema; DXA, dual‐energy X‐ray absorptiometry.

As proposed, all women were recommended to present before and during subsequent pregnancy for close clinical and biochemical monitoring of the basic treatment as well as DXA scans before and after pregnancy (Fig. 4A). All women had a natural birth and presented 2–4 weeks after delivery, as suggested. Following our management algorithm, weaning was initiated within the first weeks after delivery due to elevated bone resorption parameters in all (Table 1 and Fig. 4B). During a mean follow‐up of 23 months, no further fractures or BME occurred.

## Discussion

In this study, we present the successful skeletal management and clinical outcomes of three women with a history of PLO and pathogenic variants in *WNT1* or *LRP5* during a subsequent pregnancy. Despite the previous occurrence of PLO with multiple vertebral and peripheral fractures, no recurrence during the following pregnancy was observed after optimized basic treatment and early initiation of weaning. Together, this extends our knowledge on the clinically highly relevant question of risk for the maternal skeleton during subsequent pregnancy in PLO.

Although it is comparatively rare in general, PLO is assumed to result from a multifactorial pathomechanism, including risk factors such as duration of lactation, low vitamin D levels, anorexia, smoking,^[^ ^17^, ^18^ ^]^ and genetic variants associated with low bone mass.^[^ ^2^, ^8^, ^9^, ^19^ ^]^ Moreover, increased calcium transfer likely contributes to excessive bone resorption^[^ ^2^, ^5^ ^]^ and reduced aBMD, especially at the spine.^[^ ^8^ ^]^ In this regard, it appears plausible that women with genetic variants are more prone to manifestations of PLO, likely explaining the comparatively high frequency of a hereditary component in this distinct cohort.^[^ ^8^, ^9^, ^18^ ^]^ For clinical routine, the genetic analysis appears to be of pivotal relevance in deciphering the PLO pathomechanism, in the respective children and family members as well as in the mother.

Although many unanswered questions remain, knowledge of this clinically relevant entity has been extended in recent decades. Previously, biochemical and histomorphometric analyses had shown decreased bone turnover in women with PLO in general and compared to women with low‐energy osteoporotic fractures unrelated to pregnancy or lactation.^[^ ^18^, ^20^ ^]^ Together with the reported structural deterioration, this suggests an increased susceptibility to clinically apparent bone health consequences, especially during periods of excessive bone resorption (e.g., pregnancy or lactation). Moreover, the high proportion of detected pathogenic variants in genes involved in the WNT signaling pathway^[^ ^8^ ^]^ supports the hypothesis that women with low bone remodeling are at higher risk of PLO. In this sense, it can be assumed that women with PLO are not able to increase the rate of bone formation to the same extent as that of bone resorption and are thus impaired in their ability to maintain balanced bone turnover, for example, due to mutations in *WNT1* or *LRP5*. In conjunction with the severely limited use of bisphosphonates, the osteoanabolic agent teriparatide appears attractive and has shown good clinical and densitometric outcomes.^[^ ^11^ ^]^

Despite the small number of included individuals and general heterogeneity of the disease, the findings appear relevant as genetic variants were shown to predispose women to more severe clinical manifestations.^[^ ^8^ ^]^ Moreover, a previous report of 30 women with PLO showed a rate of 20% for new fractures in the context of subsequent pregnancies,^[^ ^14^ ^]^ and in another report of three women, one showed recurrence of PLO.^[^ ^21^ ^]^ In the present study, none of the women suffered additional fractures, and bone mass and structure were stable during the subsequent pregnancy compared to baseline. Although aBMD recovery after onset of PLO was described previously,^[^ ^3^, ^6^, ^7^ ^]^ the fact that neither aBMD nor HR‐pQCT parameters did not further decrease but remained stable or even increased after the subsequent pregnancy was not expected and underlines the great importance of optimized basic treatment. While in most cases optimal vitamin D status (i.e., above 30 μg/L) and a healthy diet are sufficient to balance calcium homeostasis and bone turnover, some individuals require additional (low‐dose) calcium supplementation, for example, when secondary hyperparathyroidism and/or elevated ALP/BALP levels exist despite optimal vitamin D status. Following general recommendations, vitamin D should be given daily during pregnancy, and lactation and hypercalcemia must be avoided. It seems particularly important that these patients be closely monitored in specialized bone centers and that the significance of timely weaning to protect the maternal skeleton be discussed already during pregnancy. In that regard, results need to be interpreted in the context of the expected changes associated with pregnancy and lactation.^[^ ^5^ ^]^

Regardless of these limitations, these new findings expand our knowledge of PLO, particularly in the relevant and relatively common group with pathogenic hereditary variants. With close monitoring, optimized treatment, and timely initiation of weaning, no recurrence of PLO, such as additional BMEs or fractures, occurred during a subsequent pregnancy. Given the limited number of individuals involved, a systematic analysis of subsequent pregnancies in women with a history of PLO and hereditary bone disease is needed to improve the management of this clinical condition.

## Author Contributions

Julian Stürznickel: Conceptualization, data curation, formal analysis, investigation, writing—original draft, and writing—review and editing. Sebastian Butscheidt: Writing—review and editing. Michael Amling: Supervision, writing—review and editing. Ralf Oheim: Conceptualization, project administration, supervision, writing—review and editing.

## Disclosures

Ralf Oheim has served as a speaker and advisory board member for Kyowa Kirin, Inozyme, Ipsen, Pharmacosmos, and UCB and received an institutional research grant from Kyowa Kirin and UCB. Julian Stürznickel, Sebastian Butscheidt, and Michael Amling state that they have no conflicts of interest.

### Peer Review

The peer review history for this article is available at https://www.webofscience.com/api/gateway/wos/peer-review/10.1002/jbm4.10779.

## Supporting information


**Fig. S1.** Densitometric evaluation. (A) Areal bone mineral density (aBMD) evaluation by dual‐energy X‐ray absorptiometry (DXA) at the lumbar spine and hip in Individual 1 (time interval BL–BP 23 months, BP–AP 10 months). (B) Three‐dimensional evaluation of bone microarchitecture by high‐resolution peripheral quantitative computed tomography (HR‐pQCT) at the distal radius in Individual 1 (time interval BL–BP 23 months, BP–AP 10 months). (C) aBMD evaluation by DXA at lumbar spine and hip in Individual 2 (time interval BL–BP 10 months, BP–AP 25 months). (D) HR‐pQCT evaluation at distal radius in Individual 2 (time interval BL–AP 35 months). (E) aBMD evaluation by DXA at lumbar spine and hip in Individual 3 (time interval BL–AP 17 months). (F) HR‐pQCT evaluation at distal radius in Individual 3 (time interval BL–AP 17 months).

## Data Availability

The data that support the findings of this study are available on request from the corresponding author. The data are not publicly available due to privacy and ethical restrictions.
