# Supplementary material for: Dynamics of Skeletal Status under Optimized Management during Subsequent Pregnancy in Three Women with a History of Pregnancy‐ and Lactation‐Associated Osteoporosis Carrying pathogenic Variants in WNT1 and LRP5
Source: JBMR Plus. 2023 Jun 21;7(8):e10779. doi: 10.1002/jbm4.10779 (PMC10443073; doi:10.1002/jbm4.10779)
Supplement: Supplementary file 1 — Fig. S1. Densitometric evaluation. (A) Areal bone mineral density (aBMD) evaluation by dual‐energy X‐ray absorptiometry (DXA) at the lumbar spine and hip in Individual 1 (time interval BL–BP 23 months, BP–AP 10 months). (B) Three‐dimensional evaluation of bone microarchitecture by high‐resolution peripheral quantitative computed tomography (HR‐pQCT) at the distal radius in Individual 1 (time interval BL–BP 23 months, BP–AP 10 months). (C) aBMD evaluation by DXA at lumbar spine and hip in Individual 2 (time interval BL–BP 10 months, BP–AP 25 months). (D) HR‐pQCT evaluation at distal radius in Individual 2 (time interval BL–AP 35 months). (E) aBMD evaluation by DXA at lumbar spine and hip in Individual 3 (time interval BL–AP 17 months). (F) HR‐pQCT evaluation at distal radius in Individual 3 (time interval BL–AP 17 months). [file JBM4-7-e10779-s001.docx]

**Dynamics of skeletal status under optimized management during subsequent pregnancy in three women with a history of pregnancy- and lactation-associated osteoporosis (PLO) carrying pathogenic variants in *WNT1* and *LRP5***

**Supplementary Figure 1: Densitometric evaluation.** (A) Areal bone mineral density (aBMD) evaluation by dual-energy X-ray absorptiometry (DXA) at the lumbar spine and hip in individual 1 (time interval BL – BP 23 months, BP – AP 10 months). (B) Three-dimensional evaluation of bone microarchitecture by high-resolution peripheral quantitative computed tomography (HR-pQCT) at the distal radius in individual 1 (time interval BL – BP 23 months, BP – AP 10 months). (C) aBMD evaluation by DXA at the lumbar spine and hip in individual 2 (time interval BL – BP 10 months, BP – AP 25 months). (D) HR-pQCT evaluation at the distal radius in individual 2 (time interval BL – AP 35 months). (E) aBMD evaluation by DXA at the lumbar spine and hip in individual 3 (time interval BL – AP 17 months). (F) HR-pQCT evaluation at the distal radius in individual 3 (time interval BL – AP 17 months).

BL – baseline, AP – after pregnancy, Tb.BMD – trabecular bone mineral density, Tb.Th – trabecular thickness, Ct.BMD – cortical bone mineral density, Ct.Th – cortical thickness.
